# Antimicrobial Susceptibility Testing in *Pseudomonas aeruginosa* Biofilms: One Step Closer to a Standardized Method

**DOI:** 10.3390/antibiotics9120880

**Published:** 2020-12-09

**Authors:** Carmen Lozano, María López, Beatriz Rojo-Bezares, Yolanda Sáenz

**Affiliations:** Área de Microbiología Molecular, Centro de Investigación Biomédica de La Rioja (CIBIR), 26006 Logroño, Spain; carmen.lozano@unirioja.es (C.L.); mlopezm@riojasalud.es (M.L.); brojo@riojasalud.es (B.R.-B.)

**Keywords:** biofilm, planktonic, resistance, beta-lactams, aminoglycosides, fluoroquinolones, azithromycin

## Abstract

The ability of *Pseudomonas aeruginosa* to form biofilm during a long-term infection makes it difficult to treat patients correctly. The current clinical antimicrobial susceptibility testing methods are based on the study of planktonic strains. A standardized protocol to analyze the antimicrobial susceptibility in biofilms is necessary for routine laboratories. The aims of this study were to develop a simple biofilm model and to study the antimicrobial susceptibility of *P. aeruginosa* strains in biofilm growth. Different artificial sputum media, and aerobiosis and microaerobiosis conditions were analyzed using a microtiter plate method and *P. aeruginosa* PAO1 as reference strain. Planktonic and biofilm antimicrobial susceptibility to cefepime, imipenem, azithromycin, gentamicin, tobramycin, and ciprofloxacin were determined in clinical and non-clinical *P. aeruginosa* strains. The Synthetic Cystic Fibrosis Medium was proposed as a good medium. The biofilm greatly increased the resistance to tested antimicrobials, except for azithromycin. Cefepime and imipenem showed poor anti-biofilm effect while tobramycin, gentamicin, and ciprofloxacin showed good activity in some strains. Azithromycin showed a better activity in biofilm than in planktonic state when aerobic conditions were used. This study establishes useful information to test antimicrobial susceptibility in *P. aeruginosa* biofilms, and includes possible antimicrobial options to treat long-term infected patients.

## 1. Introduction

*Pseudomonas aeruginosa* is an opportunistic Gram-negative pathogen with great ability to survive under a variety of environmental conditions. This microorganism is one of the most relevant causes of severe human nosocomial infections, especially in immunocompromised patients. *P. aeruginosa* colonizes very efficiently in the human respiratory tract and its prevalence is very high in patients with cystic fibrosis (CF), bronchiectasis, and chronic obstructive pulmonary disease. If *P. aeruginosa* is not eradicated during the first infection phase, this species forms biofilms in the airways. The development of biofilm, organized bacterial communities embedded in an extracellular polymeric matrix attached to surfaces, is currently recognized as one of the major determinants in persistent infections [1].

This bacterial species shows resistance to a high number of antimicrobials due to several intrinsic mechanisms and either as a consequence of mutations or by acquiring genetic material from other bacteria. The increasing prevalence of *P. aeruginosa* strains resistant to almost all classes of antipseudomonal agents has been reported [2]. Moreover, it has been observed that strains in biofilm are more resistant than those in planktonic form [3,4]. One of the major outstanding issues in routine practice is that current antimicrobial susceptibility testing methods are based on the study of planktonic strains. This approach is not adequate to determine the antimicrobial phenotype of bacteria growing in biofilms, and subsequently is not adequate to determine the correct treatment. Some biofilm susceptibility testing methods have been proposed, but a standardization of the procedures remains needed [4,5,6,7]. Moreover, some artificial media that simulate human sputum have been developed [8,9,10,11,12,13]. The use of these artificial media could reproduce more faithfully what is actually happening in the patient’s lung. However, only few studies have tested the activity of antimicrobial agents using these media [8,11,12,13]. The comparison of these media to know their possible usefulness in clinical laboratories would be highly recommended. It has been suggested that the susceptibility to some antimicrobial groups is most affected by the presence of biofilms than to others due to different tolerance mechanisms [3]. It would be interesting to have more information about the antimicrobial susceptibility in *P. aeruginosa* biofilms in order to know which antimicrobial groups are more or less efficient as treatment choice.

The aim of this study was to develop a simple biofilm model based on the use of a medium that mimics the sputum of the patients for studying *P. aeruginosa* biofilm, and to use it to analyze the biofilm antimicrobial susceptibility in a collection of seven *P. aeruginosa* strains from different origins, antimicrobial susceptibility, and biofilm capacity.

## 2. Results and Discussion

### 2.1. Minimum Metabolic Inhibitory Concentrations of P. aeruginosa PAO1

Table 1 shows Planktonic and Biofilm Minimum Metabolic Inhibitory Concentrations (P-MMIC and B-MMIC, respectively) of *P. aeruginosa* PAO1 reference strain to six tested antimicrobial agents (cefepime, imipenem, azithromycin, gentamicin, tobramycin, and ciprofloxacin) using different media and growth conditions. As expected, *P. aeruginosa* PAO1 strain was more resistant to all tested antimicrobials in biofilm than in planktonic state except in the case of azithromycin. Azithromycin is a macrolide agent that was less active in aerobic planktonic PAO1 than in aerobic biofilm PAO1 in most of the media. This antimicrobial agent is usually used as adjutant in the treatment of some chronic lung infections due to its anti-inflammatory action as a result of its capacity to decrease the immune system response [14]. Moreover, in last years, it has been analyzed its possible role as an anti-biofilm agent and it has been observed that azithromycin can significantly inhibit *P. aeruginosa* biofilm formation and motility [15].

No significant differences were observed between the P-MMIC values obtained in the four culture media, with the exception of gentamicin, tobramycin, and azithromycin. Both aminoglycosides showed higher P-MMIC values (>2 dilutions) in all artificial media in aerobic and microaerobic conditions. Azithromycin also showed higher P-MMIC values in Synthetic Cystic Fibrosis Medium (SCFM) and in a modification of SCFM (SCFM-2) when microaerobic conditions were used (≥2 dilutions) (Table 1). Differences in P-MMIC of some antimicrobials (such as tobramycin, ciprofloxacin, ceftazidime, and meropenem in *P. aeruginosa* or vancomycin, azithromycin, tobramycin, and linezolid in *Staphylococcus aureus*), have been previously described comparing cation-adjusted Mueller-Hinton broth (CAMHB) medium with other media [11,16]. However, while in our study P-MMIC to tobramycin was higher in ASM medium (4 mg/L) than in CAMHB (0.5 mg/L), in the study of Díaz-Iglesias et al., similar MIC values were obtained in both media (0.125 and 0.25 mg/L, in ASM and CAMHB medium, respectively) [11].

Regarding B-MMIC values, a greater resistance was detected under microaerobic conditions than in aerobiosis in the four media (Table 1). Similar results have been obtained by others [8,17,18]. Moreover, higher values of B-MMIC in aerobiosis were identified in Artificial Sputum Medium (ASM), SCFM, and SCFM-2 media than in CAMHB medium (increasing from one to 6-fold) (Table 1). Remarkably, B-MMIC of tobramycin in aerobiosis was similar in all tested media except in ASM in which lower activity was detected. This is in accordance with a very recently published study in which this antimicrobial agent was more active in the control medium (Trypticase soy broth supplemented with glucose and NaCl) than in ASM [11].

### 2.2. Biofilm Model: Selection of Artificial Sputum Medium and Growth Conditions

Various difficulties were detected during the preparation and preservation of the different artificial culture media (Table 2). The ASM was the most laborious to prepare, being this medium and SCFM-2 the most expensive and the most difficult to preserve.

Considering the data obtained in Table 1 and the information shown in Table 2, the SCFM was selected for the study of the remaining *P. aeruginosa* strains, although ASM and SCFM-2 seem to mimic much better the composition of the sputum of CF patients [11]. SCFM was the most suitable to prepare and to preserve, and the most economical option. These properties make this medium more suitable for its use in routine clinical laboratories. Moreover, in most cases, the data obtained with this medium were very similar to those obtained with ASM and SCFM-2 media (the difference among them was 0 to 2 dilutions) (Table 1).

Despite the fact that in all cases the values of B-MMIC of PAO1 in microaerobiosis were equal or higher than the highest concentration of antimicrobial agent used (Table 1), aerobic and microaerobic conditions were kept for the study of the clinical and non-clinical strains, because the oxygen availability is variable considering different in vivo conditions of a bacterial infection [19]. For example, the amount of oxygen is usually very limited in airways infections [20]. This oxygen limitation may reduce, in a strain-dependent manner, the susceptibility to some antimicrobials. In fact, hypoxia might cause an increased antimicrobial resistance by causing altered efflux regulation (*mexEF-oprN*, *mexCD-oprJ*), down regulation of energy metabolism, reduced drug uptake, or decreased toxic hydroxyl radical production [21,22,23,24].

In the case of *P. aeruginosa* PAO1, biofilm models were produced into 24- and 96-well plates, and the same results were obtained in both plates. For that reason, 96-well plates were selected for the study of the clinical and non-clinical strains.

### 2.3. Biofilm Antimicrobial Susceptibility of Clinical and Non-Clinical P. aeruginosa Strains

**Figure 1:**
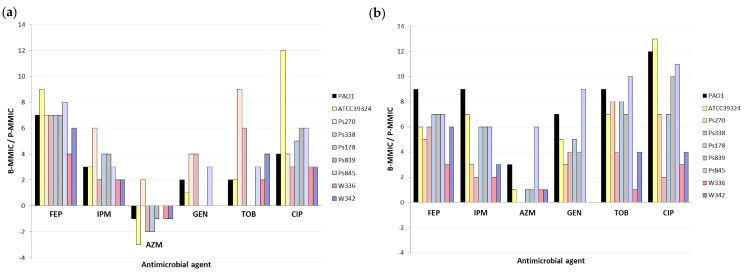
Fold change of serial twofold dilutions of Biofilm Minimum Metabolic Inhibitory Concentrations (B-MMICs) and Planktonic Minimum Metabolic Inhibitory Concentrations (P-MMICs) (B-MMIC/P-MMIC) in (**a**) aerobiosis and (**b**) microaerobiosis. FEP, cefepime; IPM, imipenem; AZM, azithromycin; GEN, gentamicin; TOB, tobramycin; CIP, ciprofloxacin.

P-MMICs and B-MMICs of the 6 antimicrobial agents were analyzed in seven *P. aeruginosa* strains from different origins, molecular typing, susceptibility, and biofilm characterization (described in Material and Methods section). Table 3 shows P-MMICs and B-MMICs detected in these strains under aerobic and microaerobic conditions. The B-MMIC/P-MMIC fold change of serial twofold dilutions is represented in Figure 1. Important differences were identified according to the antimicrobial agent tested.

The biofilm formation greatly increased the resistance to cefepime in all strains (B-MMICs 128-> 512 mg/L) (Table 3) and increased the MMIC of imipenem from 4 to >256-fold (2 to 9-fold change of serial twofold dilutions) depending on the strains (Table 3 and Figure 1). Poor anti-biofilm effect of beta-lactams has also been previously detected [4,11,25]. It is thought that this is due to the slow growth of bacteria in biofilms, and the release of beta-lactamases from killed bacteria in the outer susceptible biofilm layer. Nevertheless, it has been suggested that some carbapenems or combinations of beta-lactams and beta-lactamase inhibitors might have good in vitro activity against *P. aeruginosa* biofilms [25,26].

In the case of the aminoglycosides studied, the results were highly variable according to the strains (Table 3 and Figure 1). In some strains (Ps178 or Ps839), no differences were identified between P-MMIC and B-MMIC under aerobic conditions, whereas in other strains (Ps270 or Ps845), the MMICs increased 8->256-folds (3 to 9-fold change of serial twofold dilutions) (Table 3 and Figure 1). An explanation of these results could be a different composition of their biofilms. Even, the possible role of extracellular polysaccharides or extracellular DNA has been investigated in aminoglycoside resistance [27,28,29]. However, information about the exact role of various matrix components in biofilm-associated antimicrobial resistance is still very limited. Another possible justification might be the possible insertional inactivation of ABC transport systems in these strains that could render their biofilms susceptible to these antimicrobials [30].

On the other hand, the biofilm production increased the ciprofloxacin resistance, although this increase was also very different depending on the strain (MMIC increased from 4 to >2048-folds) (2 to 13-fold change of serial twofold dilutions) and, as expected, was higher in microerobiosis than in aerobiosis (Table 3 and Figure 1). In other studies, B-MMIC of ciprofloxacin in *P. aeruginosa* was very similar to the planktonic MIC, being considered good anti-biofilm agents [4,25]. It is important to remark that this anti-biofilm effect seems to decrease in conditions of low oxygen concentrations [31]. Indeed, it has been observed that oxygenation by hyperbaric oxygen treatment increases the bactericidal activity of ciprofloxacin on *P. aeruginosa* biofilm [32].

Finally, as we observed in *P. aeruginosa* PAO1, azithromycin showed greater activity in biofilm than in planktonic state of all clinical and no-clinical strains, except for Ps270 strain, corroborating its possible role as anti-biofilm agent (Figure 1a). Nevertheless, this effect was only detected in aerobic conditions, since equal or lower activity was detected in biofilm in microaerobiosis (Figure 1b). Taking into consideration these results, the lack of oxygen might cause that this antimicrobial loses its anti-biofilm action.

The higher resistance values detected to most of the antimicrobial agents under microaerobiosis than aerobiosis conditions could be due to the increased biofilm production. To analyze this hypothesis, the bacterial metabolic activity was determined inside the biofilm structure formed in SCFM during 72 h under aerobic or microaerobic conditions in absence of antimicrobial agents (Table 4). However, in many strains, the metabolic activity within the biofilm formed with low oxygen concentration was very similar or even lower than in aerobic conditions. The slow growth of this bacterium or the use of alternative metabolic pathways, among other possible mechanisms, could cause this increased resistance in microaerobiosis [4,21,22,25], which requires more studies.

## 3. Materials and Methods

### 3.1. Biofilm Model Development

A standardized biofilm model that could be used in routine clinical laboratories was searched. For that, among available quantification biofilm techniques, a microtiter plate method was chosen due to its simplicity [4]. Plates of 24 and 96 wells and an initial 10^6^ cfu/mL inoculum of *P. aeruginosa* PAO1 reference strain were used. Different artificial media that simulate human sputum were tested: Artificial Sputum Medium (ASM), Synthetic Cystic Fibrosis Medium (SCFM), and a modification of SCFM (SCFM-2) [8,9,10]. These media were compared with cation-adjusted Mueller-Hinton broth (CAMHB) medium, which is the recommended medium for antimicrobial susceptibility testing [33]. An initial 10^6^ cfu/mL inoculum of *P. aeruginosa* PAO1 strain was added to 24- and 96-well plates of using 1.8 and 0.18 mL per well, respectively. Plates were incubated for 72 h under aerobic or microaerobic (5% O_2_ and 10% CO_2_; using Campygen^TM^ sachets in Rectangular AnaeroBoxTM, OXOID) conditions at 37 °C and shaking at 75 rpm to obtain a mature biofilm.

A total of 200 μL (in 24-well plates) or 20 μL (in 96-well plates) of serial twofold dilutions of different antimicrobials were added to each well with the formed biofilm (concentration range in mg/L): cefepime (FEP) (0.25–512), imipenem (IPM) (0.06–128), azithromycin (AZM) (0.25–512), gentamicin (GEN) (0.125–256), tobramycin (TOB) (0.125–256), and ciprofloxacin (CIP) (0.25–512). All experiments were performed in triplicate wells for each antimicrobial, and repeated at least three times. After 24 h of incubation in the presence of the antimicrobial agents, 100 μL (in 24-well plates) or 10 μL (in 96-well plates) of cellulase (100 mg/mL, diluted in 0.05 M citrate buffer) was used for disrupting the bacterial biofilms as previously described [8]. Fluorescein diacetate (FDA) and resazurin dyes were tested in order to measure the viability of the cells in the biofilm [34]. For that, 100 μL (in 24-well plates) or 10 μL (in 96-well plates) of FDA (0.2 mg/L) or resazurin (0.02% *v*/*v*) were added to each well, and incubated for 1 h at 37 °C in the dark. Fluorescent measures using an excitation/emission wavelength of 494/518 nm (FDA) or 540/590 nm (resazurin) were performed using a POLARstar Omega microplate reader (BMG Labtech) [8,35]. When using FDA, some problems were detected in some artificial media, such as ASM, because high fluorescent values were detected in the control wells (without bacteria). Possibly, FDA interacts with some components of these media and, for that reason, resazurin was used in the remaining experiments.

Biofilm Minimum Metabolic Inhibitory Concentrations (B-MMICs) of FEP, IPM, AZM, GEN, TOB, and CIP were determined in *P. aeruginosa* PAO1 reference strain using ASM, SCFM, and SCFM-2 artificial media and CAMHB medium. B-MMICs were defined as the antimicrobial concentrations causing 90% inhibition of metabolic activity in biofilm [8].

Planktonic Minimum Metabolic Inhibitory Concentrations (P-MMICs) of the same antimicrobial agents were also determined using the same three artificial media (ASM, SCFM, SCFM-2) and CAMHB medium. An initial 10^6^ cfu/mL inoculum of *P. aeruginosa* PAO1 strain was added to 96-well plates, and subsequently exposed to serial twofold dilutions of the antimicrobial agents. After 24 h of incubation, 10 μL of resazurin (0.02% *v*/*v*) were added and incubated for 1 h at 37 °C in the dark, and after that, the reaction was measured. P-MMICs were defined as the antimicrobial concentrations causing 90% inhibition of metabolic activity in planktonic form.

All B-MMIC and P-MMIC experiments were performed in triplicate wells for each antimicrobial agent, and repeated at least three times.

### 3.2. Biofilm Antimicrobial Susceptibility of Clinical and Non-Clinical P. aeruginosa Strains

Seven clinical (isolated from clinical samples of patients in hospital settings) and non-clinical (obtained from food samples) *P. aeruginosa* strains were selected from the *Pseudomonas* collection of the Molecular Microbiology group (Center for Biomedical Research of La Rioja, CIBIR, Spain), by their susceptibility to all tested antimicrobial agents, their capacity to produce biofilm, and by belonging to international or high-risk clones (Table 5). The selection of these strains was intended to analyze, in a biofilm model, the behavior of a small collection of strains adapted or disseminated to different environments. The criteria included different origins (sputum, bronchial aspirate, wound, and food), molecular typing (ST155, ST235, ST412), antimicrobial susceptibility (multidrug and moderate resistant, susceptible), and biofilm capacity (hyper and low producers). Two control strains were also included, *P. aeruginosa* PAO1 reference strain and *P. aeruginosa* ATCC39324 strain. P-MMICs and B-MMICs of FEP, IPM, AZM, GEN, TOB, and CIP in the seven clinical and non-clinical *P. aeruginosa* strains and the two control strains were determined using the selected SCFM artificial medium and the resazurin dye. For biofilm antimicrobial susceptibility, mature biofilms were obtained after 72 h under aerobic and microaerobic conditions. All experiments were performed in triplicate wells for each condition, and repeated at least three times.

Moreover, the metabolic activity inside the biofilm produced using SCFM medium under aerobic or microaerobic conditions after 72 h of incubation was quantified for the seven clinical and non-clinical *P. aeruginosa* strains. The biofilm metabolic activity was determined without presence of antimicrobial agents by using FDA staining in all strains [34,35]. Measures were also performed using a POLARstar Omega microplate reader (BMG Labtech). The assays were performed in triplicate.

## 4. Conclusions

In conclusion, SCFM seems to be a good artificial medium for obtaining a future standard method to study biofilm susceptibility in clinical routine laboratories. In the case of airways infections, in which low oxygen availability is present, microaerobic conditions are highly important to be considered to test planktonic and biofilm *P. aeruginosa* susceptibility. Some antimicrobial agents such as cefepime or imipenem seem to be very inefficient in biofilms, indicating that maybe these agents should be avoided in long-term infected patients. However, tobramycin, gentamicin, and ciprofloxacin seem to have higher activity than other antimicrobials and could be a good option. It would be necessary to test the biofilm activity of each case since a high variability of results was identified according to the strain tested. Finally, azithromycin showed a better activity in biofilm than in planktonic state but only when aerobic conditions were used.

## Figures and Tables

**Table 1:** Planktonic and Biofilm Minimum Metabolic Inhibitory Concentrations of *P. aeruginosa* PAO1 reference strain using different culture media and growth conditions ^1^.

Antimicrobial	P-MMIC (mg/L)			B-MMIC (mg/L)	
	Aerobiosis	Microaerobiosis		Aerobiosis	Microaerobiosis
	CAMHB/ASM/ SCFM/SCFM-2	CAMHB/ASM/ SCFM/SCFM-2		CAMHB/ASM/ SCFM/SCFM-2	CAMHB/ASM/ SCFM/SCFM-2
Cefepime	1/1/1/2	0.5/1/1/2		32/64/128/512	>512/>512/512/>512
Imipenem	1/0.5/1/0.5	1/0.5/0.5/1		4/32/8/16	>128/>128/>128/>128
Azithromycin	8/8/16/16	32/32/128/256		0.25/2/8/16	512/>512/>512/>512
Gentamicin	0.5/4/4/8	0.5/4/4/8		4/16/16/16	>256/>256/>256/>256
Tobramycin	<0.125/0.5/0.5/1	0.125/0.5/1/2		2/8/2/2	>256/>256/>256/>256
Ciprofloxacin	<0.25/<0.25/<0.25/0.25	<0.25/<0.25/<0.25/0.5		<0.25/0.5/2/4	>512/>512/512/>512

^1^ Abbreviations: P-MMIC, Planktonic Minimum Metabolic Inhibitory Concentrations; B-MMIC, Biofilm Minimum Metabolic Inhibitory Concentrations; CAMHB, cation-adjusted Mueller-Hinton broth; ASM, Artificial Sputum Medium; SCFM, Synthetic Cystic Fibrosis Medium; SCFM-2, SCFM supplemented with salmon sperm DNA, GlcNAc, bovine maxillary mucin, and dioleoylphosphatidylcholine. All experiments were performed in triplicate wells for each condition and repeated at least three times.

**Table 2:** Preparation, preservation, and cost of the artificial media used in this study.

Artificial Medium ^1^	Time of Preparation	Time of Preservation	Cost
ASM	2 days + at least 2 days of filtration	1 month	++
SCFM	1 day	2 months	+
SCFM-2	3 days	1 month	++

^1^ ASM, Artificial Sputum Medium; SCFM, Synthetic Cystic Fibrosis Medium; SCFM-2, SCFM supplemented with salmon sperm DNA, GlcNAc, bovine maxillary mucin, and dioleoylphosphatidylcholine.

**Table 3:** P-MMICs and B-MMICs of different antimicrobial agents against the clinical and non-clinical *P. aeruginosa* strains using SCFM artificial medium ^1^.

Strain	MLST	P-MMIC (mg/L)/B-MMIC (mg/L)												
		Aerobiosis							Microaerobiosis					
		FEP	IPM	AZM	GEN	TOB	CIP		FEP	IPM	AZM	GEN	TOB	CIP
PAO1	ST549	1/128	1/8	16/8	4/16	0.5/2	<0.25/2		1/512	0.5/>128	128/>512	4/>256	1/>256	<0.25/512
ATCC39324	-	2/>512	2/16	64/8	8/16	2/8	<0.25/512		16/>512	2/>128	512/>512	16/>256	4/>256	<0.25/>512
Ps270	ST412	8/>512	4/>128	256/>512	32/>256	1/>256	2/32		32/>512	32/>128	>512/>512	64/>256	2/>256	4/>256
Ps338		4/512	1/4	128/32	4/64	0.5/32	1/8		8/512	1/4	512/512	4/64	2/32	2/8
Ps178	ST155	8/>512	2/32	32/8	16/16	2/2	2/64		8/>512	4/>128	512/>512	16/>256	2/>256	4/512
Ps839		8/>512	1/16	128/64	32/32	4/4	<0.25/8		8/>512	4/>128	512/>512	32/>256	4/>256	0.5/512
Ps845		4/>512	1/8	8/8	0.25/2	0.25/2	<0.25/8		8/>512	4/>128	16/>512	1/>256	0.5/>256	<0.25/256
W336	ST235	64/>512	64/>128	32/16	>256/>256	128/>256	64/512		128/>512	64/>128	512/>512	>256/>256	256/>256	128/>512
W342		16/>512	16/64	32/16	>256/>256	4/64	64/512		16/>512	32/>128	512/>512	>256/>256	8/128	64/>512

^1^ Abbreviations: MLST, multilocus sequence typing; P-MMIC, Planktonic Minimum Metabolic Inhibitory Concentrations; B-MMIC, Biofilm Minimum Metabolic Inhibitory Concentrations. FEP, cefepime; IPM, imipenem; AZM, azithromycin; GEN, gentamicin; TOB, tobramycin; CIP, ciprofloxacin. All experiments were performed in triplicate wells for each condition, and repeated at least three times.

**Table 4:** Metabolic activity of bacteria within the biofilm formed during 72 h at 37 °C in SCFM medium under aerobic and microaerobic conditions determined by fluorescein diacetate staining method.

Strain	Biofilm Metabolic Activity (as % Compared to PAO1) ± SD ^1^	
	Aerobiosis	Microaerobiosis
ATCC39324	265 ± 3	138 ± 5
Ps270	375 ± 12	404 ± 8
Ps338	281 ± 9	175 ± 4
Ps178	730 ± 2	275 ± 16
Ps839	319 ± 9	448 ± 6
Ps845	247 ± 6	391 ± 3
W336	232 ± 18	241 ± 4
W342	164 ± 6	378 ± 6

^1^ All assays were performed in triplicate. The data are expressed as % mean ± standard deviation (SD).

**Table 5:** Information about the seven clinical and non-clinical *P. aeruginosa* strains, and the *P. aeruginosa* PAO1 and ATCC39324 control strains selected for this study.

Strain	Origin	MLST	Antimicrobial Susceptibility ^1^	Biofilm (%) ± SD ^2^	
				Biomass (CV) ^3^	Metabolic Activity (FDA) ^4^
PAO1	Control	ST549		100 ± 4	100 ± 8
ATCC39324	Control (Sputum CF patient)	-		83 ± 12	13 ± 14
Ps270	Sputum CF patient	ST412	IPM, MEM, DOR, FEP, CAZ, TZP, GEN, AMK, NET, LEV	301 ± 20	222 ± 10
Ps338	Sputum CF patient	ST412	Susceptible	92 ± 17	5 ± 18
Ps178	Sputum	ST155	Susceptible	267 ± 2	454 ± 18
Ps839	Food (swiss chard)	ST155	IMP	208 ± 34	694 ± 95
Ps845	Food (swiss chard)	ST155	Susceptible	511 ± 119	802 ± 93
W336	Bronchial aspirate	ST235	IPM, MEM, DOR, FEP, CAZ, TZP, GEN, CIP	49 ± 14	65 ± 10
W342	Wound	ST235	IPM, MEM, GEN, CIP	55 ± 14	101 ± 12

^1^ Determined by disc diffusion method. IPM, imipenem; MEM, meropenem; DOR, doripenem; FEP, cefepime; CAZ, ceftazidime; TZP, piperacillin-tazobactam; GEN, gentamicin; AMK, amikacin; NET, netilmicin; LEV: levofloxacin; CIP, ciprofloxacin. ^2^ Biofilm (as % compared to PAO1) were obtained after bacterial incubation for 24 h under aerobic conditions at 37 °C without shaking and using Mueller-Hinton medium. All assays were performed in triplicate. The biofilm metabolic activity is expressed as % mean ± standard deviation (SD). ^3^ Crystal violet stain method [35]. ^4^ Fluorescein diacetate stain method [35].
